# Colorectal cancer-derived small extracellular vesicles induce TGFβ1-mediated epithelial to mesenchymal transition of hepatocytes

**DOI:** 10.1186/s12935-023-02916-8

**Published:** 2023-04-18

**Authors:** Marzia Pucci, Marta Moschetti, Ornella Urzì, Marco Loria, Alice Conigliaro, Maria Antonietta Di Bella, Rossella Crescitelli, Roger Olofsson Bagge, Alessia Gallo, Mark F. Santos, Caterina Puglisi, Stefano Forte, Aurelio Lorico, Riccardo Alessandro, Simona Fontana

**Affiliations:** 1 Department of Biomedicine, Neurosciences and Advanced Diagnostics, University of Palermo, Palermo, Italy; 2 Sahlgrenska Center for Cancer Research, Department of Surgery, Institute of Clinical Sciences, Sahlgrenska Academy, University of Gothenburg, Gothenburg, Sweden; 3 Wallenberg Centre for Molecular and Translational Medicine, University of Gothenburg, Gothenburg, Sweden; 4 Department of Surgery, Sahlgrenska University Hospital, Gothenburg, Sweden; 5 Department of Research, IRCCS ISMETT, Palermo, Italy; 6 Touro University College of Medicine, Henderson, NV USA; 7 IOM Ricerca, Viagrande, Catania Italy; 8 Institute for Biomedical Research and Innovation (IRIB), National Research Council (CNR), Palermo, Italy

**Keywords:** Colorectal cancer, Small extracellular vesicles, Transforming growth factor‑β1 (TGFβ1), Hepatocytes, Liver metastasis

## Abstract

**Background:**

Metastatic disease is the major cause of cancer-related deaths. Increasing evidence shows that primary tumor cells can promote metastasis by preparing the local microenvironment of distant organs, inducing the formation of the so-called “pre-metastatic niche”. In recent years, several studies have highlighted that among the tumor-derived molecular components active in pre-metastatic niche formation, small extracellular vesicles (sEVs) play a crucial role. Regarding liver metastasis, the ability of tumor-derived sEVs to affect the activities of non-parenchymal cells such as Kupffer cells and hepatic stellate cells is well described, while the effects on hepatocytes, the most conspicuous and functionally relevant hepatic cellular component, remain unknown.

**Methods:**

sEVs isolated from SW480 and SW620 CRC cells and from clinical samples of CRC patients and healthy subjects were used to treat human healthy hepatocytes (THLE-2 cells). RT-qPCR, Western blot and confocal microscopy were applied to investigate the effects of this treatment.

**Results:**

Our study shows for the first time that TGFβ1-carrying CRC_sEVs impair the morphological and functional properties of healthy human hepatocytes by triggering their TGFβ1/SMAD-dependent EMT. These abilities of CRC_sEVs were further confirmed by evaluating the effects elicited on hepatocytes by sEVs isolated from plasma and biopsies from CRC patients.

**Conclusions:**

Since it is known that EMT of hepatocytes leads to the formation of a fibrotic environment, a well-known driver of metastasis, these results suggest that CRC_sEV-educated hepatocytes could have an active and until now neglected role during liver metastasis formation.

**Supplementary Information:**

The online version contains supplementary material available at 10.1186/s12935-023-02916-8.

## Background

Metastatic disease is the major complication in the clinical management of most types of cancer, and it is the cause of cancer-related deaths even more than the primary tumor [1]. Thus, the importance of identifying new key molecular and cellular components driving the complex steps of the metastatic process is crucial to develop new strategies to prevent and control this fatal evolution of tumor disease. Increasing evidence shows that primary tumor cells can promote metastasis by preparing the local microenvironment of distant organs even before their arrival, inducing the formation of the so-called “pre-metastatic niche” (PMN), a permissive and fertile “soil” on which cancer cells may grow, ultimately promoting tumor metastasis [2]. In recent years, several studies have highlighted that among the tumor-derived molecular components active in initiating PMN formation, small extracellular vesicles (sEVs) play a crucial role. Small EVs are nanometer-sized extracellular vesicles (30–200 nm diameter) released by all cell types under both physiological and pathological conditions that function as mediators of cellular crosstalk, delivering to target cells a broad array of selected and biologically active molecules (proteins, microRNAs, mRNAs, long non-coding RNAs, lipids and metabolites). The small size and relative stability allow sEVs to transit from their site of origin to serum as well as in other biofluids, including cerebrospinal fluid, saliva, and urine, thus affecting the behavior and phenotype of both neighboring and distant cells and influencing a broad variety of cellular activities in health and disease [3–5]. The biological role of sEVs has been widely studied in cancer. Tumor cells actively produce, release, and utilize sEVs, creating a communication network that promotes tumor progression and metastasis. Tumor-derived sEVs (TD_sEVs) transfer information locally within the tumor microenvironment as well as systemically to distant tissue sites, where they can induce the shaping of the PMN, functioning as key signals between primary tumor cells and future metastatic sites [6, 7]. Several studies have demonstrated the ability of TD_sEVs to modulate PMN formation in the liver, which after lymph nodes, is the organ most frequently afflicted by metastasis. The liver has a complex cellular composition that includes parenchymal cells, namely, hepatocytes and cholangiocytes, and non-parenchymal cells, such as liver sinusoidal endothelial cells, hepatic stellate cells, Kupffer cells, and liver-associated lymphocytes. During hepatic metastatic processes, the properties of this cellular microenvironment, which also includes recruited inflammatory and immune cells, determine the response to invading cancer cells. In recent years, a number of studies have highlighted the crucial role played by TD_sEVs in initiating liver PMN formation, specifically affecting the activities of Kupffer cells, hepatic stellate cells, and recruited macrophages [7–9], while the involvement of hepatocytes remains unknown. Even if this cell component is the most conspicuous in the liver and is responsible for several physiological activities of this complex organ, its role in liver metastasis is partly described and related to the pro-metastatic process [10]. No clear data about the TD_sEV-mediated enrollment of hepatocytes during pre-metastatic niche formation are currently available. Deeper knowledge of the involvement of educated TD_sEV hepatocytes in preparing tumor secondary sites could contribute to better understanding and defining how the liver promotes and supports metastasis growth. Costa‐Silva and coworkers showed that EVs released by pancreatic cancer cells enriched in migration inhibitory factor (MIF) induce Kupffer cells to secrete transforming growth factor β1 (TGFβ1), which elicits the profibrotic activity of hepatic stellate cells supporting the recruitment of bone marrow-derived macrophages and finally priming the liver for pancreatic cancer metastasis [11]. Recent studies have reported that the liver fibrosis niche is a favorable microenvironment for metastatic formation and that the liver fibrosis score is a powerful prognostic factor for CRC hepatic metastasis and relapse [12, 13]. Interestingly, even if hepatic stellate cells are widely considered the main matrix-producing cells driving liver fibrosis, recent studies have also described a TGFβ1/SMAD-dependent EMT of hepatocytes associated with the processes of hepatic fibrosis, a key condition for liver metastatic priming [14–16]. In our study, we showed that sEVs derived from CRC cells (CRC_sEVs) trigger TGFβ1/SMAD-mediated EMT in healthy hepatocytes. Since it is known that EMT may lead hepatocytes to induce fibrosis, an essential component of liver metastasis [17], the findings of this study will open new avenues for both basic and translational cancer research, pointing out hepatocyte activities as new potential targets for designing and developing novel therapies for the prevention or early treatment of liver metastasis.

## Methods

### Cell cultures and small extracellular vesicle isolation from the cell supernatant

The SV40 large T antigen-immortalized healthy human liver epithelial cell line THLE-2 (ATCC, Manassas, VA) [18] was cultured in Airway Epithelial Cell Basal Medium (ATCC, Manassas, VA) with the Bronchial Epithelial Cell Growth Kit (ATCC PCS-300–040, Manassas, VA) supplemented with 70 ng/ml phosphoethanolamine, 5 ng/ml epidermal growth factor (EGF), 10% fetal bovine serum, 100 U/mL penicillin, and 100 μg/ml streptomycin (Euroclone, UK) at 37 °C with 5% CO_2_. Cells were maintained in precoated flasks with a collagen coating made of a mixture of 0.01 mg/ml fibronectin (Sigma-Aldrich, St Louis, MO, USA), 0.03 mg/ml bovine collagen type I (Advanced Biomatrix, San Diego region, California, USA), and 0.01 mg/ml bovine serum albumin (Sigma-Aldrich, St Louis, MO, USA).

The colorectal cancer cell lines SW480 (ATCC CCL-228) and SW620 (ATCC CCL-227) are isogenic cell lines derived from pre-metastatic primary tumors and lymph node metastasis, respectively. They were maintained in RPMI 1640 medium (Euroclone, UK) supplemented with 10% fetal bovine serum (FBS; Euroclone UK), 2 mM l-glutamine (Euroclone, UK), 100 U/ml penicillin, and 100 µg/ml streptomycin (Euroclone, UK). Before use, FBS (South America origin EU approved, Euroclone, UK) was ultracentrifuged for 105 min at 100,000 × *g* in a Type 70 Ti fixed angle rotor to eliminate bovine sEVs.

Small EVs were isolated from the conditioned culture medium of SW480 and SW620 cells maintained in the presence of EV-depleted FBS. The conditioned medium was collected after a culture period of 24 h and then subjected to differential centrifugation followed by ultracentrifugation as previously described [19]. Briefly, the conditioned culture medium was centrifuged for 5 min at 300 × g, 15 min at 3,000 × g and 30 min at 10,000 × g; the supernatant was then ultracentrifuged for 105 min at 100,000 × g in a Type 70 Ti fixed angle rotor. The obtained sEV pellet was resuspended in a range of 50–100 μl PBS, quantified by the Bradford protein assay (Pierce, Rockford, IL, USA) [20] and stored at − 80 °C until further use. Moreover, the concentrations and size distribution of sEVs were measured by Nanoparticle Tracking Analysis (NTA) (NanoSight NS300, Malvern Instruments Ltd, UK). Samples were diluted in phosphate buffered saline (PBS) 1:100 to reach the optimal concentration for instrument linearity. The particle size measurement was calculated on a particle-by-particle basis in 3 videos of 60 s to provide accuracy and statistics for each analysis under the following conditions: cell temperature: 23.3°-23.6 °C; syringe speed: 30 µl/s. Recorded data were analyzed for the mean, mode, median, and estimated concentration of particles by the in-build NanoSight Software NTA 3.3 with a detection threshold of 5. Hardware: embedded laser: 45 mW at 488 nm; camera: sCMOS [21].

### Small extracellular vesicle isolation from human clinical samples

The results obtained by using sEVs from cell cultures were validated by using sEVs isolated from the plasma of CRC patients and healthy donors (Table 1) and from human biopsies (Table 2). Following the manufacturer’s instructions, a Total Exosome Isolation Kit (Invitrogen) was used to isolate sEVs from 100 μl of plasma samples. Briefly, the plasma was centrifuged for 20 min at 2000 × g and 20 min at 10,000 × g at room temperature. The supernatant was mixed with 50 μl of 1X PBS and vortexed, and then 30 μl of exosome precipitation reagent (from plasma) was added to the samples and vortexed again. The samples were incubated at room temperature for 10 min and centrifuged for 5 min at 10,000 × g at room temperature. The pellets of sEVs were resuspended in 1X PBS, and the protein content was determined by the Bradford assay (Pierce, Rockford, IL, USA). At the end of the isolation procedure, the sEV pellets obtained from plasma samples were resuspended in a range of 50–100 μl PBS, quantified by the Bradford protein assay (Pierce, Rockford, IL, USA) [20], and stored at − 80 °C until further use. Particle size distribution and concentration were measured as described in the previous paragraph.

**Table 1:** Information about plasma samples from patients (P) with colon cancer and healthy controls (HS)

Plasma sample	Sex	Age	Disease status	Diagnosis	TNM-stage	Grading
P1	F	48	CRC	ADC	T3N0Mx	2
P2	M	75	CRC	ADC	T3N1aMx	2
P3	M	71	CRC	ADC	T3N0Mx	2
P4	M	74	CRC	ADC	T3N0Mx	2
P5	M	55	CRC	ADC	T3N2bMx	2
HS1	M	57	Absent	–	–	–
HS2	F	44	Absent	–	–	–
HS3	F	26	Absent	–	–	–
HS4	F	35	Absent	–	–	–
HS5	F	39	Absent	–	–	–

*CRC* Colorectal Cancer; *ADC* Adenocarcinoma

**Table 2:** Information about biopsy samples of patients with CRC

Biopsy	Sex	Age	Diagnosis	TNM-stage	Grading
NCRC/B1	M	79	ADC	T3bN0Mx	3
CRC/B1					
NCRC/B2	M	58	ADC	T3N2aMx	3
CRC/B2					

*CRC/B* Colorectal Cancer biopsy; *NCRC/B* Non-Colorectal Cancer mucosa biopsy; *ADC* Adenocarcinoma

Small EVs were also isolated from CRC biopsy (CRC/B, Table 2) and from Non-CRC mucosa (Non-CRC biopsies: NCRC/B, Table 2) of the same patient using the protocol established previously by Crescitelli R. et al. [22] with minor modifications. The tissue pieces were gently sliced into small fragments (1–2 mm) and incubated with collagenase D (Roche, Basel, Switzerland) (2 mg/ml) and DNase I (Roche, Basel, Switzerland) (40 U/ml) dissolved in RPMI plain medium (St Louis, MO, USA) for 30 min at 37 °C. After incubation, the samples were passed through a 70 µm filter. The resulting filtered liquid was centrifuged at 300 × g for 10 min and 2,000 × g for 20 min and ultracentrifuged at 16,500 × g for 6 min (TLA 100.3, k-factor: 404.5, Beckman Coulter, Miami, FL, USA) to remove cells, tissue debris, and large EVs. The remaining supernatant was ultracentrifuged at 120,000 × g for 65 min (TLA 100.3, k-factor: 55.5, Beckman Coulter) to pellet sEVs. The pellet was resuspended in PBS and further purified by a bottom-loaded iodixanol density cushion (OptiPrepTM, Sigma-Aldrich, St Louis, MO, USA). Briefly, the sEVs were bottom-loaded by mixing a 1 ml sample with 3 ml of 60% OptiPrepTM that was placed at the bottom of an ultracentrifuge tube. On top of this, 4 ml of 30% OptiPrepTM and 4 ml of 10% OptiPrepTM were carefully layered on top. The samples were then centrifuged at 97,000 × g for 2 h (SW 41 Ti, k-factor: 265.1, Beckman Coulter, Miami, FL, USA). After centrifugation, the visible band containing the purified vesicles was collected from the 10%/30% interface (1.078 g/mL and 1.175 g/ml OptiPrepTM). To remove the contamination from OptiPrepTM, sEVs were further centrifuged at 120,000 × g for 65 min (TLA 100.3, k-factor: 55.5, Beckman Coulter, Miami, FL, USA) [22]. Small EV proteins were estimated with the Qubit assay system (Thermo Fisher Scientific, Waltham, MA, USA) following the manufacturer’s instructions.

### Transmission electron microscopy (TEM)

To perform morphological characterization of isolated sEVs, TEM microscopy analyses were performed in two different labs depending on the origin of the processed samples. Since the protocols used presented some slight differences, we report both. The sEV samples were negatively stained before acquisition.

1. For sEVs isolated from conditioned culture medium and plasma samples, sEVs were prepared for electron microscopy studies using negative staining. Approximately 5 μl of the sEV suspension was deposited onto carbon-coated EM grids. After washing, the samples were fixed for 5 min in 1% glutaraldehyde and negatively stained with a 2% aqueous solution of phosphotungstic acid. The grids were viewed in a JEM 1400 Plus electron microscope (Jeol, Japan) operating at 80 kV equipped with a CCD camera.

2. For sEVs isolated from tissue samples, sEVs were investigated by negative staining as previously described [22]. Briefly, 2.3 μg of sEVs was placed onto glow-discharged 200-mesh formvar/carbon copper grids (Electron Microscopy Sciences, Hatfield Township, PA). After two washes in H_2_O, sEVs were fixed in 2.5% glutaraldehyde. After two further washes in H_2_O, the samples were stained with 2% uranyl acetate for 1.5 min. Negative-stained samples were examined on a digitized Talos L120C electron microscope (Thermo Fisher Scientific) at 120 kV with a CCD camera.

### Treatment of hepatocytes

To analyze the effects induced by CRC_sEVs on hepatocytes, the following treatment protocol was applied. After reaching subconfluence, THLE-2 cells were treated for the indicated time points with approximately 1.5*E10 particles of sEVs derived from SW480 and SW620 cell lines corresponding to 20 μg/ml, the dose we found effective in our previous study [19]. Thus, the same number of sEVs/ml was used for treating THLE-2 cells with sEVs from the plasma of healthy subjects and CRC patients or from CRC and adjacent non-CRC biopsies. After treatment, the cells were harvested for real-time quantitative PCR, Western blot or immunofluorescence analysis by confocal microscopy.

### Labeling and internalization of CRC_sEVs

To evaluate the ability of hepatocytes to directly interact with CRC_sEVs, an uptake experiment was performed. For this purpose, SW480_sEVs were labeled with PKH26 according to the manufacturer’s instructions. Briefly, sEVs were incubated with PKH26 for 10 min at room temperature, washed in PBS by three centrifugations, resuspended in growth medium and finally incubated with THLE-2 cells for 3 and 6 h at 37 °C. After incubation, cells were stained with ActinGreenTM 488 Ready ProbesR Reagent (Life Technologies, USA), which binds F-actin with high affinity, with Hoechst (Molecular Probes, Life Technologies, USA) to stain nuclei and observed by confocal microscopy (Nikon A1).

### Cytotoxicity assay

To evaluate whether sEVs can have a toxic effect on hepatocyte growth, the CellTox™ Green Cytotoxicity Assay was performed (G8741, Promega, Madison, WI, USA). THLE-2 cells were cultured in triplicate at 5 × 10^3^ cells/well in white-walled, opaque 96-well plates; 24 h post-seeding, cells were treated for 24 and 48 h with approximately 1.5*E10 particles of SW480_sEVs, SW620_sEVs, CRC_P/sEVs (P1-5) and HS/sEVs (HS1-5). Changes in membrane integrity that occur as a result of cell death were measured as relative fluorescence units (RFUs) by Glomax (Promega, Madison, WI, USA).

### Western blot

THLE-2 cells or sEVs (from cells or plasma) were lysed using RIPA buffer with protease inhibitor cocktail (Thermo Fisher Scientific, Waltham, MA, USA) (1:100 dilution) for 30 min on ice and then centrifuged at 18,800 × g for 15 min at 4 °C. The extracted proteins were measured using the Bradford protein assay (Pierce, Rockford, IL, USA). Proteins were separated on Bolt 4–12% Bis–Tris Plus precast polyacrylamide gels (Invitrogen by Thermo Fisher Scientific, Waltham, MA, USA) under reducing conditions, except for TGFβ1. Following electrophoresis, proteins were transferred to a nitrocellulose blotting membrane (Amersham Protran Premium 0.45 µm NC by GE HealthCare Life Science, Little Chalfont, Buckinghamshire, UK), blocked in 1% BSA and incubated with primary antibodies overnight at 4 °C. Primary antibodies used for sEV characterization [23] were: anti-CD81 (1:1000 dilution; Santa Cruz Biotechnology, Dallas, TX, USA), anti-HSC70 (1:1000 dilution; Santa Cruz Biotechnology, Dallas, TX, USA), anti-Calnexin (1:1000; Santa Cruz Biotechnology, Dallas, TX, USA), anti-cytochrome c (1:1000; Cell Signaling Technology, Danvers, MA, USA); the primary antibody anti-TGFβ1 antibody (1:300 dilution; Santa Cruz Biotechnology, Dallas, TX, USA) was used to assess the presence of TGFβ1 in CRC_sEVs; primary antibodies used for investigating the modulation of mediators of the TGFβ1 signalling and the related targets were: anti-SMAD 2/3 (1:300 dilution; Cell Signaling Technology, Danvers, MA, USA), anti-pSMAD 2/3 (1:300 dilution; Cell Signaling Technology, Danvers, MA, USA), anti-SNAIL (1:300 dilution; Cell Signaling Technology, Danvers, MA, USA), anti-SLUG (1:300 dilution; Cell Signaling Technology, Danvers, MA, USA), anti-Vimentin (1:300 dilution; Cell Signaling Technology, Danvers, MA, USA), anti-αSMA (1:300 dilution; Cell Signaling Technology, Danvers, MA, USA), anti-CK8/18 (1:300 dilution; Cell Signaling Technology, Danvers, MA, USA), anti-E-Cadherin (1:300 dilution; Cell Signaling). Anti-β actin (1:1000 dilution; Santa Cruz Biotechnology, Dallas, Tx, USA), anti-GAPDH (1:1000 dilution; Santa Cruz Biotechnology, Dallas, TX, USA), and anti-Tubulin (1:1000 dilution; Santa Cruz Biotechnology, Dallas, TX, USA) were employed to detect proteins used as loading control. After washing with Tris-buffered saline + Tween 20 (TBS/T) three times, the membrane was incubated with horseradish peroxidase (HRP)-conjugated goat anti-rabbit or anti-mouse secondary antibodies (1:1000 dilution; Thermo Fisher Scientific, Waltham, MA, USA) at room temperature for 1 h. The protein bands were visualized by enhanced chemiluminescence (ECL™ Prime Western Blotting System Cytiva RPN2232) by using the Chemidoc imaging system (Bio-Rad, Milan, Italy). Densitometric analysis of the Western blot was performed by using ImageJ software.

### Real-time PCR

Total RNA was extracted using an illustra™ RNA spin mini-RNA isolation kit (GE Healthcare, Little Chalfont, Buckinghamshire, UK). The RNA was reverse transcribed to cDNA using the High-Capacity cDNA Reverse Transcription kit (Applied Biosystems, Foster City, CA, USA). Then, the cDNA was subjected to quantitative real-time reverse transcriptase-polymerase chain reaction (RT-PCR) analysis. The sequences of the primers used are reported in Table 3. Real-time PCR was performed using a Step One™ Real-time PCR System Thermal Cycling Block (Applied Biosystems, Waltham, MA, USA) in a 20 μl reaction containing 300 nM of each primer, 2 μl template cDNA, and 18 μl 2X SYBR Green I Master Mix. The PCR was run at 95 °C for 20 s followed by 40 cycles of 95 °C for 3 s and 60 °C for 30 s. GAPDH was used as the endogenous control. Relative changes in gene expression between control and treated samples were determined using the ΔΔCt method.

**Table 3:** Primers used in RT-PCR

Primers	Forward	Reverse
*GAPDH*	*ATGGGGAAGGTGAAGGTCG*	*GGGTCATTGATGGCAACAATAT*
*ALB*	*GAGACCAGAGGTTGATGTGATG*	*GCCATCATCTTCTTTGACCCA*
*APOE*	*TGGCACTGGGTCGCTTTTGGG*	*TCATGGTCTCGTCCATCAGCGC*
*CYP3A4*	*AAGTCGCCTCGAAGATACACA*	*AAGGAGAGAACACTGCTCGTG*

### ELISA

The amount of albumin in the culture medium of THLE-2 cells treated with SW480- and SW620-derived sEVs was determined by a human albumin ELISA kit (Abcam, Cambridge, UK). ELISA was performed according to the manufacturer’s instructions.

### Confocal fluorescence microscopy

After the indicated treatment, the cells were fixed by the addition of 4% PAF, permeabilized with 0.1% Triton X-100 and incubated for 1 h at r.t. with the following primary antibodies: anti-HNF4 (1:50 dilution; Santa Cruz Biotechnology, Dallas, TX, USA), anti-CK8/18 (1:50 dilution; Cell Signaling Technology, Danvers, MA, USA), and anti-Vimentin (1:50 dilution; Cell Signaling Technology, Danvers, MA, USA). Unbound primary antibody was then removed, and the cells were washed with ice-cold PBS and incubated with DyLight 488 or DyLight 594 secondary antibody (1:500 dilution; Thermo Fisher Scientific, Waltham, MA, USA). Unbound secondary antibody was removed, cells were washed with ice-cold PBS, and nuclei were stained with Hoechst (Molecular Probes, Life Technologies, Carlsbad, CA, USA). In some cases, cells were stained with Actin Green (Thermo Fisher, Waltham, MA, USA) (1:125 dilution) to stain F-actin. Finally, the samples were analyzed by confocal fluorescence microscopy (Nikon A1 confocal microscope).

### dSTORM characterization

Direct stochastic optical reconstruction microscopy (dSTORM) is an emergent single-molecule super-resolution imaging technique with a practical resolution limit of 20 nm used extensively to image and characterize the anatomy, organization, and biomechanical properties of subcellular structures as EVs [24]. The sEVs prepared from SW480 or SW620 cells (1.8 × 10^7^ or 3.5 × 10^7^ particles, respectively) were immunolabeled overnight at 4 °C using a cocktail of fluorescently labeled antibodies against CD9 (Atto 488 mouse anti-human monoclonal; FL-REA-EV-CD9-Atto 488, ONI), CD63 (Cy3BTM mouse anti-human monoclonal; FL-REA-EV-CD63-Cy3b, ONI), and TGFβ1 (Alexa Fluor® 647 mouse anti-human monoclonal; IC10502R, R&D Systems). They were then loaded and captured on the surface of a PEG-biotin functionalized microfluidic chip included in the EasyVisi Single-Extracellular Vesicle Characterization kit from ONI (beta version 1.0, Oxford Nanoimaging, UK). Surface preparation, removal of unbound antibodies, and crosslinking of sEVs to the chip surface, including all wash steps, were performed using the EasyVisi kit and automated using a Roboflow microfluidic sample preparation platform (ONI). dSTORM imaging was then performed after freshly prepared BCubed STORM imaging buffer (ONI) was added to each lane on the microfluidics chip. Single-molecule fluorescence data consisting of 2000 frames per channel were sequentially acquired using the Nanoimager S Mark II with laser power set to 45, 50, and 50% for the 640, 560, and 488 lasers, respectively. An Olympus 1.4NA 100 × oil immersion super apochromatic objective was used with the angle of illumination set to 52.5°. Channel mapping was calibrated at the start of the imaging session using 0.1 µm Tetraspeck beads (#T7279, Thermo Fisher Scientific). Data were processed using NimOS software (version 1.18; ONI). To identify EV subpopulations that express one, two, or three markers, single-molecule data were analyzed using algorithms developed by ONI via their online localization microscopy data analysis platform beta-released named CODI (https://alto.codi.bio/, releases 0.16.0 to 0.14.1; March 9th to April 28th, 2021). The analysis workflow of sEV data included filtering, drift correction, and subsequent clustering using hierarchical density-based clustering algorithms for single-EV analysis.

### Trypsin digestion of CRC_sEVs and TGFβ1 ELISA

To assess the EV surface localization of TGFβ1**,** approximately 200 μg of SW480_sEVs and SW6200_sEVs were resuspended in a final volume of 0.5 ml of PBS with 0.125% trypsin (Corning, Manassas, VA) and incubated at 37 °C for 15 min under agitation [25, 26]. At the end of the reaction, the sEVs were diluted in 40 ml PBS and subjected to ultracentrifugation for 105 min at 100,000 × g in a Type 70 Ti fixed angle rotor. Finally, the pellet was resuspended in PBS and analyzed by NTA to assess the integrity of sEVs after treatment with trypsin and by ELISA to verify TGFβ1 removal. Untreated SW480_sEVs and SW620_sEVs were used as a control.

The presence of TGFβ1 in trypsin-treated and untreated SW480_sEVs and SW6200_sEVs was determined by using a TGFβ1-specific ELISA kit (Sigma-Aldrich, USA). The assay was performed using the same number of particles determined by NTA. The ELISA assay was then performed according to the manufacturer’s instructions.

### Statistical analysis

Statistical analysis was performed using GraphPad Prism software 9.5.1 (GraphPad software, Inc., La Jolla, CA). Values reported in all graphs are the mean ± standard deviation (SD) of three replicates, unless otherwise stated. The statistical significance of the differences was analyzed using a two-tailed Student’s t-test. A p-value ≤ 0.05 was considered significant.

## Results

### CRC_sEVs modulate the expression of hepatocyte markers

**Fig. 1:**
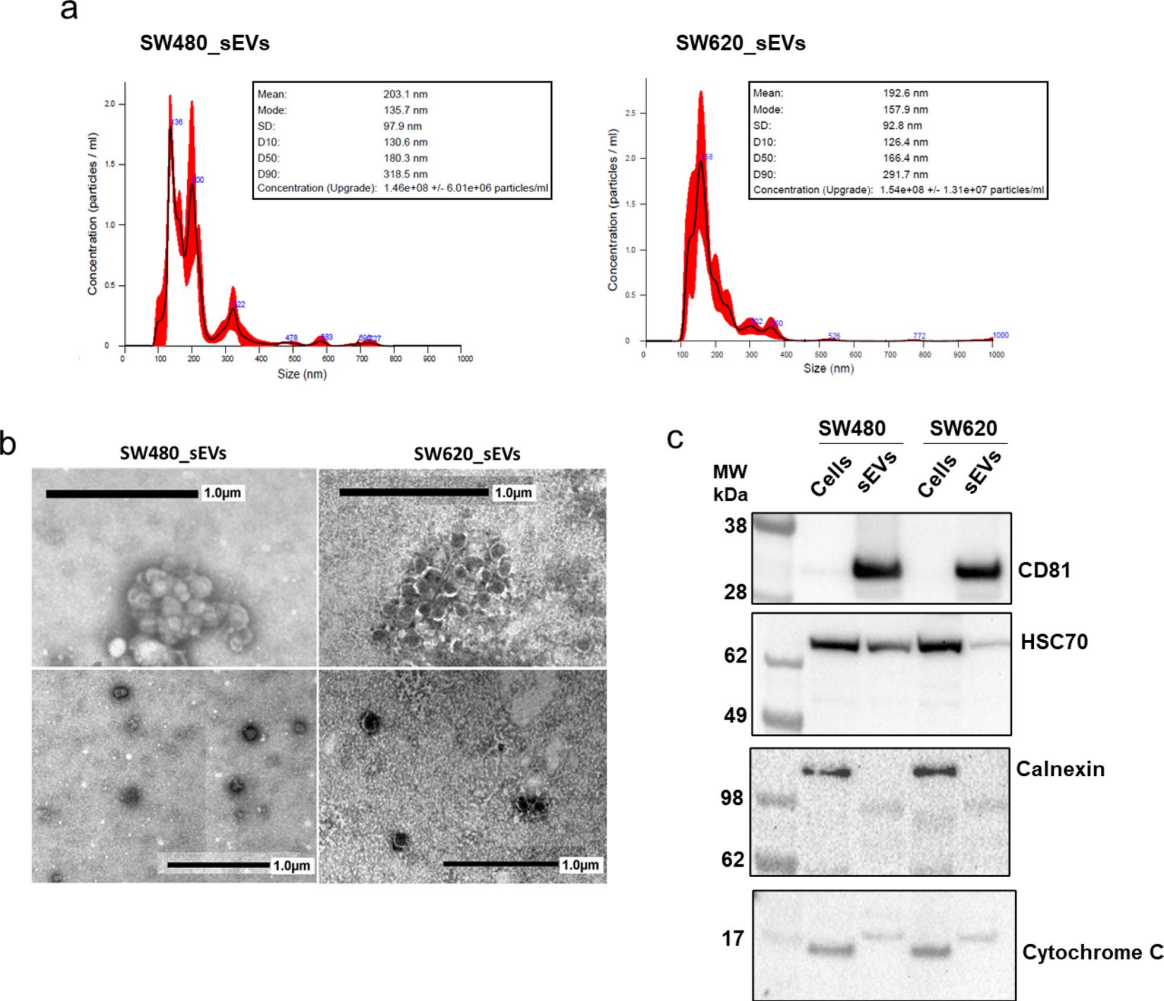
Characterization of sEVs isolated from CRC cell-conditioned media by ultracentrifugation. **a** NTA of the sizes of SW480-derived sEVs (SW480_sEVs) and SW620-derived sEVs (SW620_sEVs). **b** Representative TEM micrographs of sEVs isolated from CRC cell culture medium in which clusters (upper panel) and single vesicles (lower panel) are shown. **c** sEVs were compared to whole cell lysates by Western blot for the presence of EV markers

Small EVs from culture supernatants of the human CRC cell lines SW480 and SW620 were isolated and collected as described in the Methods section and characterized. NTA showed that sEVs isolated from SW480 cells were more heterogeneous in size than those derived from SW620 cells, but the two populations had equivalent concentrations of 1.5 × 10^8^/ml (Fig. 1a) (corresponding videos are shown in Additional File 1 and Additional file 2). Regarding the observed size, we found that both SW480_sEVs and SW620_sEVs have an average size close to what was previously described, [23] as confirmed by the modal size (Fig. 1a). Next, the TEM images reported in Fig. 1b showed particles with the typical spherical structure mainly ranging between 120–150 nm in accordance with the classical sEV size distribution, confirming the NTA results. Finally, sEVs derived from CRC cell lines were characterized by protein analysis. Typical EV markers (HSC70 and CD81) were positive in sEVs, while the absence of Calnexin and Cytochrome C indicated no contamination by endoplasmic reticulum and mitochondrial proteins (Fig. 1c).

**Fig. 2:**
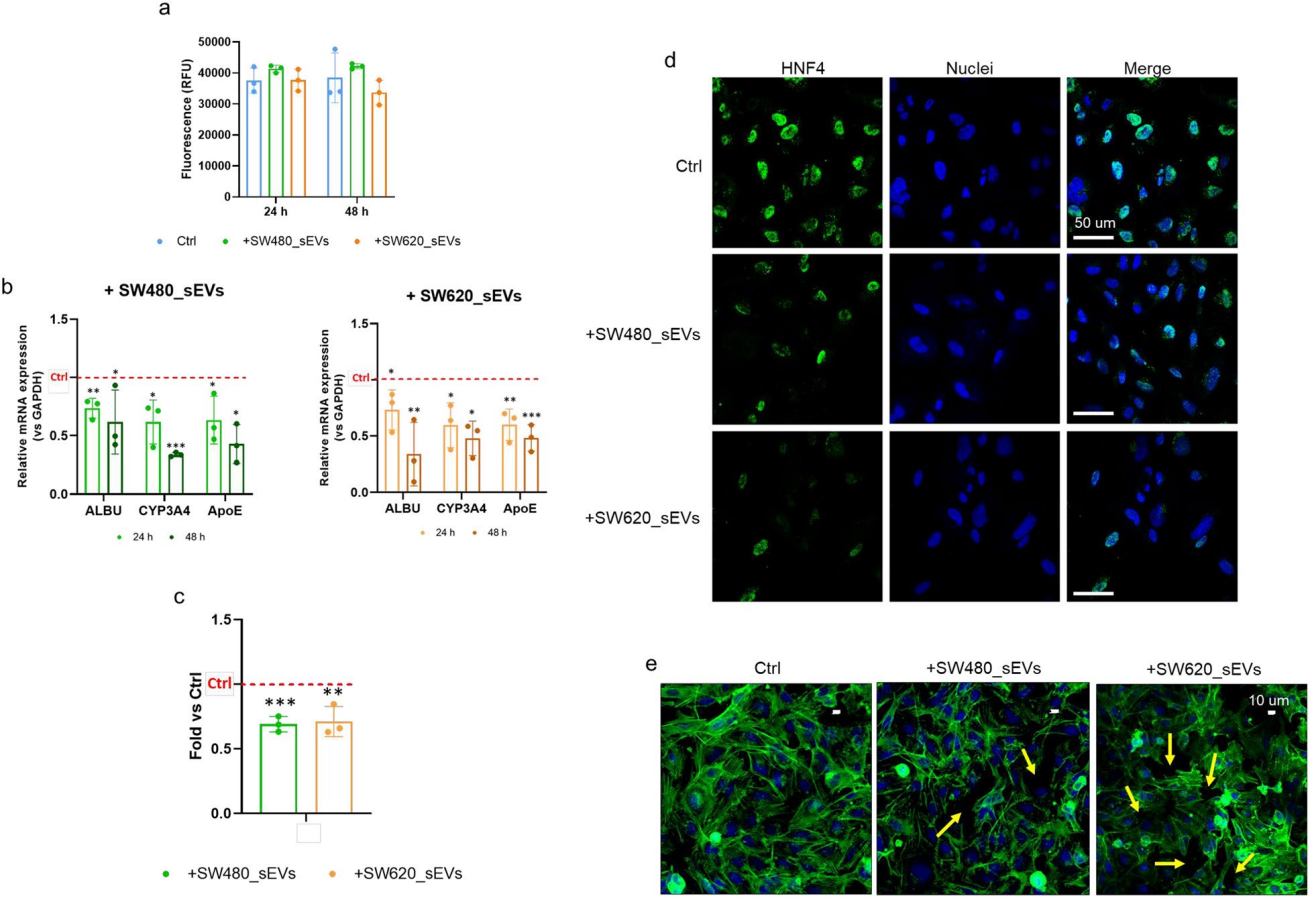
CRC_sEVs alter the functional properties and morphology of hepatocytes. **a** CellTox assay showed that treatment for 24 and 48 h with CRC_sEVs did not alter the viability of hepatocytes (RFU: relative fluorescence units). **b** Gene expression levels of ALBU, CYP3A4, and APOE were measured in THLE-2 cells treated with CRC_sEVs. **c** ELISA of ALBU released in the conditioned medium of hepatocytes treated with CRC_sEVs for 24 h. In all reported graphs, the asterisks indicate significant differences vs untreated control cells (Ctrl) (*p < 0.05; **p < 0.01; ***p < 0.01). **d** Fluorescent confocal microscope images showing HNF4 expression and localization in hepatocytes treated for 24 h with CRC_sEVs; **e** Fluorescent confocal microscope images showing the morphological changes induced by treatment with CRC_sEVs for 24 h; Actin Green (green) was used to stain actin fibers; Hoechst (blue) was used to stain the nuclei; the yellow arrows indicate the CRC_SEV-induced holes in the hepatocyte monolayer. *Ctrl* untreated control cells

After ensuring that CRC_sEVs have no toxic effects on hepatocytes (Fig. 2a) and evaluating their ability to interact with CRC_sEVs by uptake experiments (Additional File 3: Figure S1), we analyzed the expression levels of some functional hepatocyte-specific genes, such as albumin (ALBU), apolipoprotein E (APOE), and cytochrome P450 3A4 (CYP3A4) [27–29], after treatment with CRC_sEVs. We observed that both SW480_sEVs and SW620_sEVs after 24 h significantly inhibited the expression of the three genes, and this effect was stronger after 48 h (Fig. 2b). Moreover, for both sEV types, the downregulation of ALBU after 24 h of treatment was also confirmed at the protein level by ELISA (Fig. 2c). To assess the ability of CRC_sEVs to alter the key phenotypic characteristics of hepatocytes, using confocal microscopy, we also analyzed the expression and localization of hepatocyte nuclear factor 4 (HNF4), a central regulator of hepatocyte differentiation and function [30]. According to the data on ALBU, APOE, and CYP3A4, we found that treatment for 24 h with CRC_sEVs induced a clear reduction in HNF4 corresponding to its low nuclear localization in comparison to control cells (Fig. 2d). It was interesting to observe that the negative modulations at the gene/protein levels corresponded to evident morphological changes. As shown in the representative micrographs in Fig. 2e, we observed that when treated with CRC_sEVs, the hepatocytes formed a monolayer that lost its regular and ordered structure and was characterized by the appearance of spaces between cells (yellow arrows).

**Fig. 3:**
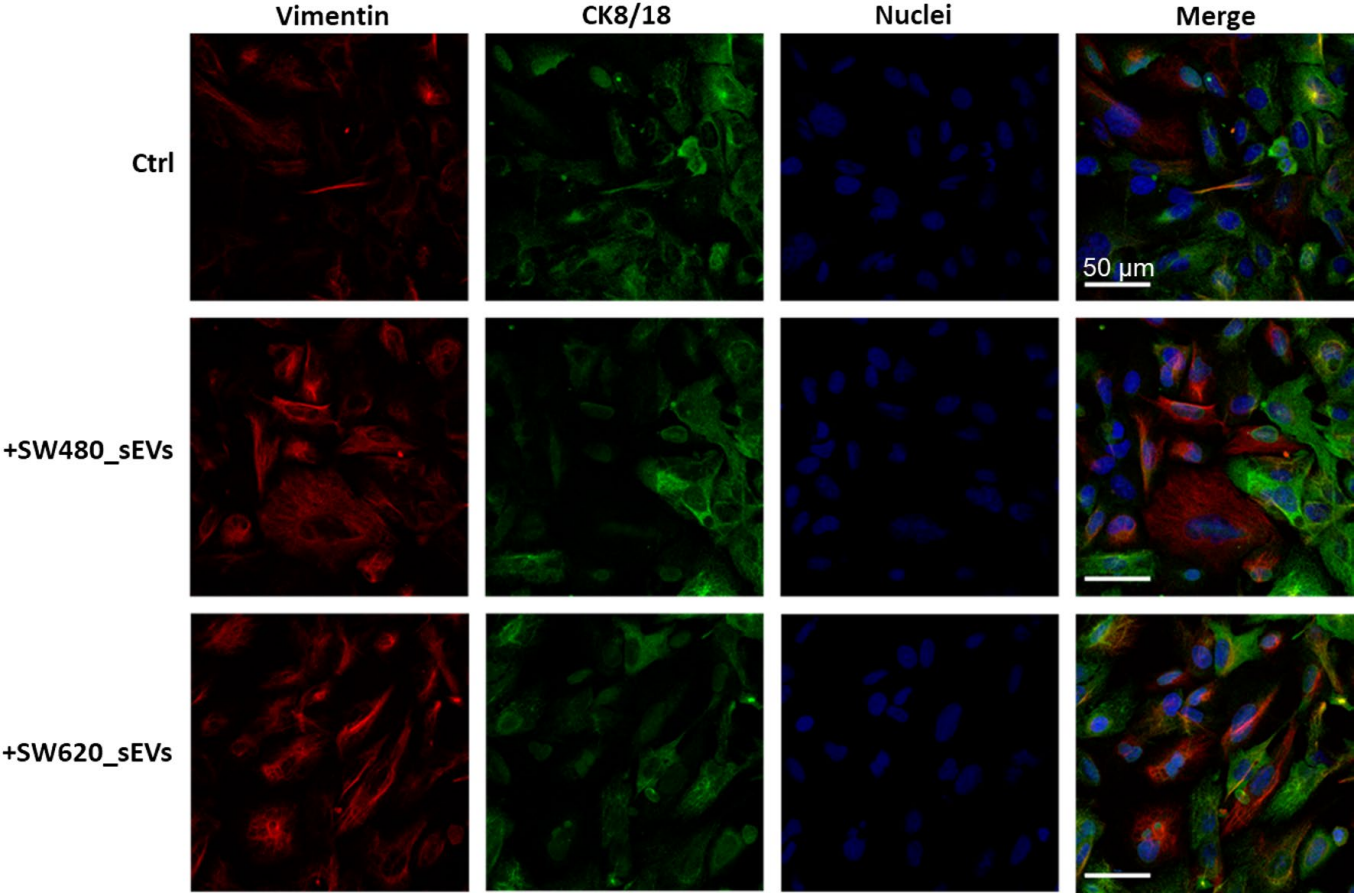
Confocal microscopy analysis of Vimentin (red) and CK8/18 (green) in hepatocytes treated with CRC_sEVs for 24 h; nuclei are in blue. Untreated cells are indicated as a control (Ctrl)

To understand whether the morphological changes observed in the CRC_sEV-treated hepatocytes were associated with modulation of structural proteins, we evaluated **t**he expression levels of the cytoskeletal proteins Vimentin and CK8/18 by confocal microscopy. The representative micrographs reported in Fig. 3 show that 24 h treatment with CRC_sEVs induced a simultaneous increase in Vimentin and decrease in CK8/18 in comparison to the control, leading us to hypothesize the ability of CRC_sEVs to initiate hepatocytes towards the EMT process.

### CRC_sEVs carrying TGFβ1 modulate the expression of EMT markers in hepatocytes

**Fig. 4:**
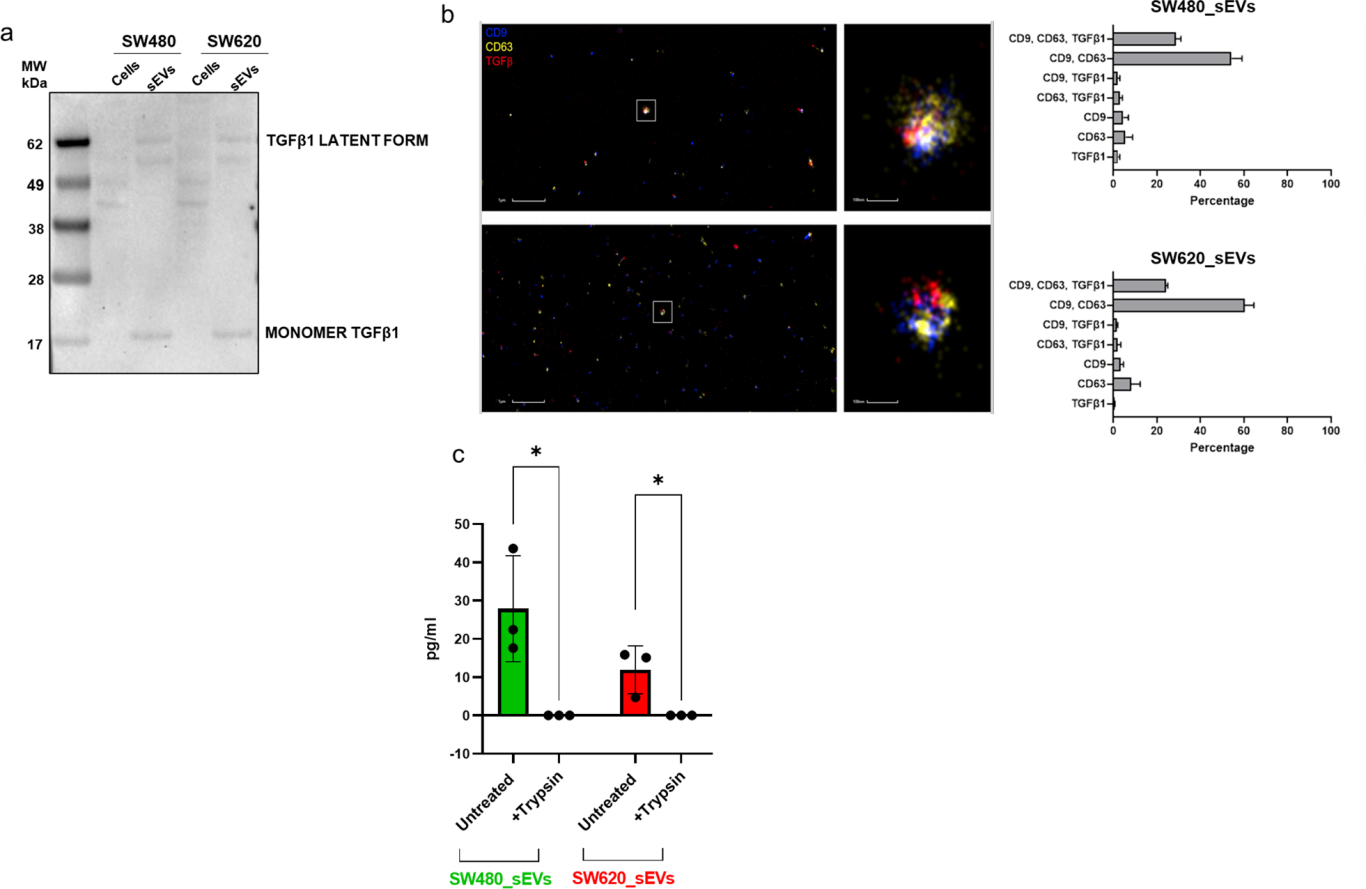
CRC_sEVs carry TGFβ1 and induce TGFβ1 signaling pathways in hepatocytes. **a** Western blot analysis of TGFβ1 in SW480 and SW620 cells and derived sEVs. **b** dSTORM imaging of SW480_ and SW620_sEVs. The graphs on the right report the ratios of the number of each sEV group to the total number of counted sEVs expressed as a percentage. **c** ELISA showing the effect of trypsin treatment on the presence of TGFβ1 in CRC_sEVs

The inhibition of ALBU and HNF4 expression, as well as the observed morphological changes associated with the modulation of Vimentin and CK8/18, can be ascribed to TGFβ1 activity [9, 31, 32], which is known to be carried by EVs, including those released by colon cancer cells [25, 33, 34]. Based on these considerations, we investigated the presence of this cytokine in sEVs isolated from both SW480 and SW620 cells. As shown in Fig. 4a, Western blot assays showed that CRC_sEVs were enriched in TGFβ1 compared with the cells, indicating that colorectal cancer cells secrete TGFβ1 into the surrounding environment through sEVs. According to data from the literature [25], we found that both sEV populations, in addition to the monomer, also carried the latent form of TGFβ1 (Fig. 4a). Furthermore, dSTORM imaging revealed that TGFβ1 is located on the surface of the sEVs, as demonstrated by its colocalization with CD9 and CD81 (Fig. 4b). Interestingly, this analysis also highlighted that both SW480 and SW620 cells release a heterogeneous population of sEVs, as already described in other cell models [35]. As reported in the graphs in Fig. 4b, we found the following seven sEV phenotypes for each cell line: CD9^+^/CD63^+^/TGFβ1^+^ (28.6% and 24%, respectively, for SW480 and SW620 cells), CD9^+^/CD63^+^ (54.3% and 60%, respectively, for SW480 and SW620 cells), CD9^+^/TGFβ1^+^ (2% and 1.6%, respectively, for SW480 and SW620 cells), CD63^+^/TGFβ1^+^ (3% and 2%, respectively, for SW480 and SW620 cells), CD9^+^ (4.5% and 3.3%, respectively, for SW480 and SW620 cells), CD63^+^ (5.6% and 8.2%, respectively, for SW480 and SW620 cells), and TGFβ1^+^ (2% and 0.5%, respectively, for SW480 and SW620 cells). These data indicated that in total, approximately 35% of sEVs released by SW480 cells and 30% of those released by SW620 cells carry TGFβ1.

To confirm the sEV surface localization of TGFβ1, we verified the effects of treatment with trypsin on the TGFβ1/sEV association. Thus, CRC_sEVs were subjected to trypsin digestion, and after assessing their integrity in comparison to untreated CRC_sEVs (Additional File 4: Figure S2), the presence of TGFβ1 was detected by ELISA. As shown in Fig. 3c, a significant decrease in TGFβ1 was observed in trypsin-treated sEVs in comparison to untreated sEVs.

**Fig. 5:**
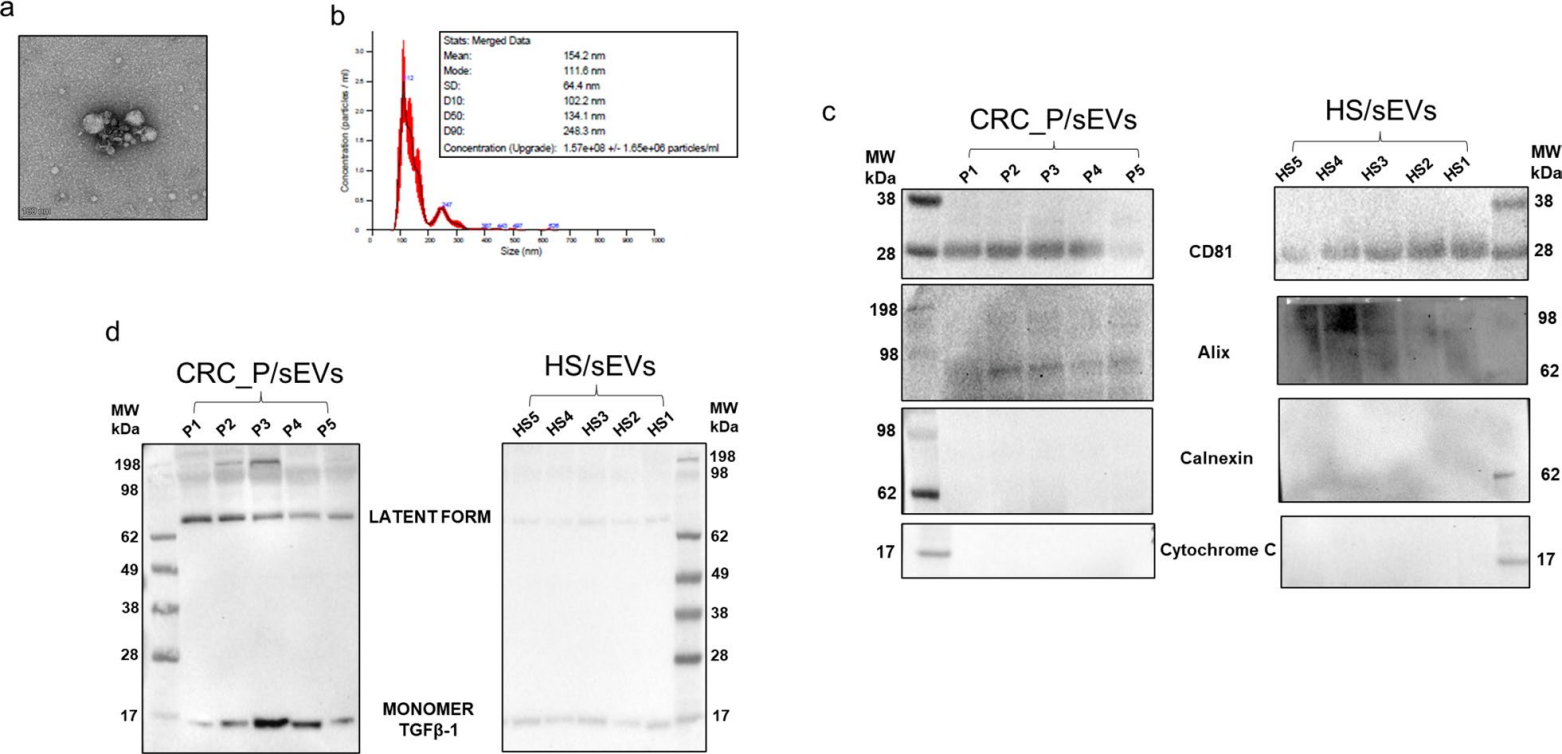
Characterization of sEVs isolated from the plasma of CRC patients (CRC_P/sEVs) and healthy subjects (HS/sEVs). Representative TEM micrograph (**a**) and NTA (**b**) of sEVs isolated from a CRC patient plasma. **c** Western blot analysis of EV markers (CD81 and Alix) and of proteins expected to be underrepresented in EVs (Calnexin and Cytochrome C). **d** Western blot analysis of TGFβ1 in CRC_P/sEVs and HS/sEVs

To corroborate our investigations, in addition to sEVs obtained by the in vitro systems, THLE-2 cells were treated with sEVs isolated from the plasma of CRC patients (CRC_P/sEVs, n = 5) and healthy subjects (HS/sEVs, n = 5). These ex vivo sEVs were characterized by TEM and NTA, and representative images (relative to the sEVs isolated from sample P2—Table 1) are reported in Fig. 5a and Fig. 5b, respectively. The video of the NTA is shown in Additional File 5 Moreover, as shown in Fig. 5c, all sEV samples obtained from the plasma of both CRC patients (P1-5) and healthy subjects (HS1-5) were characterized for the presence of EV markers (CD81 and Alix) and for the absence of proteins expected to be underrepresented in EVs (Calnexin and Cytochrome C). Finally, Western blot assays showed an appreciable difference in the amount of the detected latent form of TGFβ1 between CRC_P/sEVs and HS/sEVs (Fig. 5d). It is important to emphasize that since we received plasma samples from CRC patients and healthy subjects at different times, Western blot assays were performed independently. Thus, we paid much attention to ensuring the same experimental conditions for a proper comparison of the obtained results. Thus, to detect TGFβ1, the same protein amount (50 μg) of each sample was loaded, and both membranes were exposed for 1 min. The two membranes stained with Ponceau showing the same protein loading are reported in (Additional file 6: Figure S3). The non-cytotoxicity of the ex vivo sEVs on hepatocytes was assessed by the CellTox assay (Additional file 7: Figure S4).

**Fig. 6:**
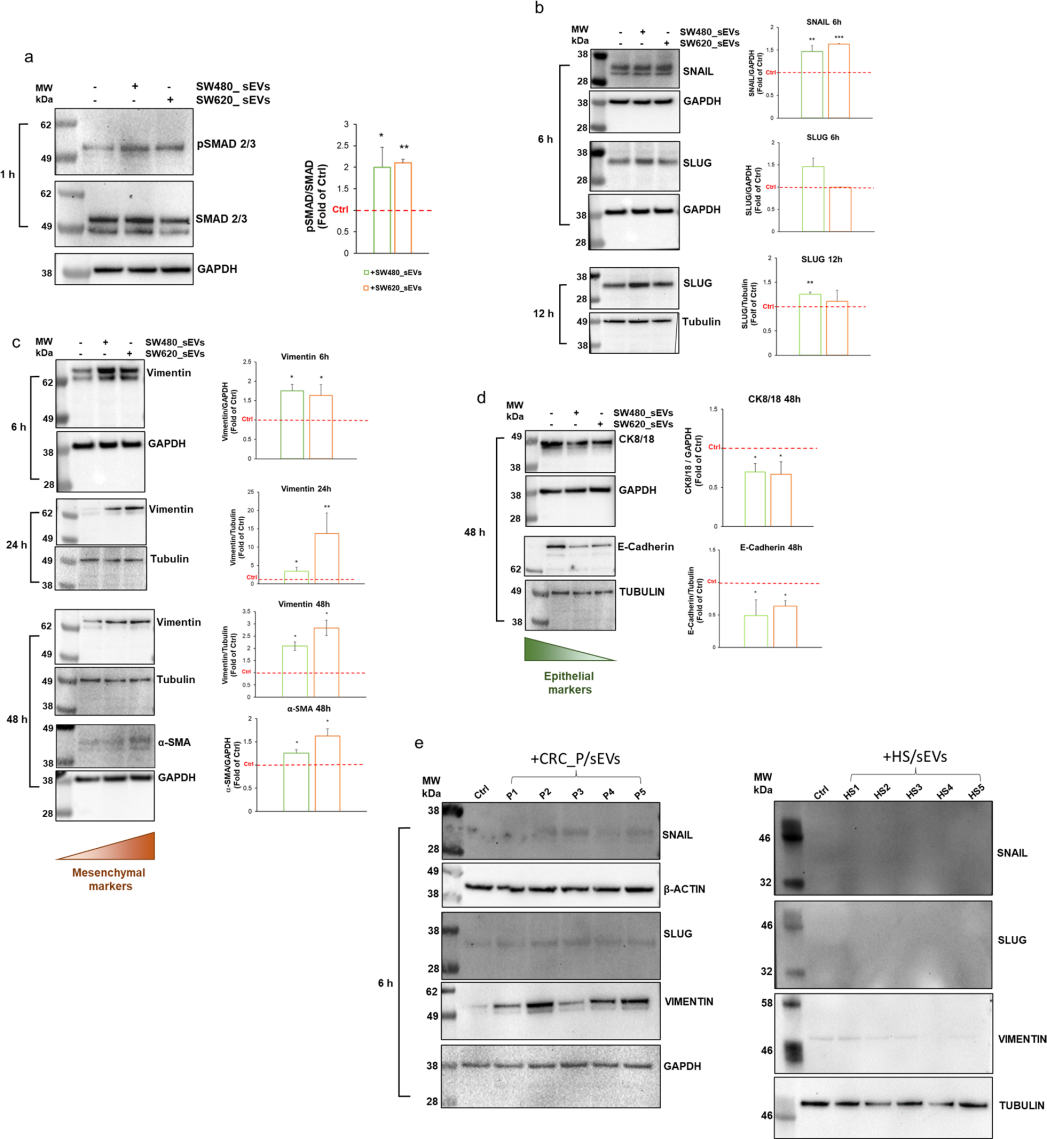
CRC_sEVs induce the modulation of TGFβ/SMAD targets associated with EMT in hepatocytes. Western blot assays were performed to verify the ability of SW480_ and SW620_sEVs to modulate SMAD 2/3 phosphorylation in hepatocytes (**a**), the expression of SNAIL and SLUG (**b**), and mesenchymal (**c**) and epithelial markers (**d**). Each Western blot is associated with the corresponding densitometric analysis where the reported values are the mean of at least 2 independent experiments (± SD) of the protein normalized vs loading control (Tubulin or GAPDH). **e** Western blot analyses of SNAIL, SLUG, and Vimentin in hepatocytes treated with CRC_P/sEVs and HS/sEVs; *Ctrl*: untreated control cells. *p ≤ 0.05; **p ≤ 0.01

Next, to further validate the biological function of TGFβ1/sEVs, we investigated the signaling pathways activated in CRC_sEV-treated hepatocytes. TGFβ1 activates a canonical signalling pathway mediated by SMAD [36]. According to the timing reported in a recent paper by Lötvall’s group [25], we found an increase in phospho-SMAD2/3 levels in hepatocytes after 1 h of treatment with CRC_sEVs (Fig. 6a), suggesting their ability to activate the canonical TGFβ1 signaling pathway. Since several studies have described the roles of TGFβ1-activated SMADs in EMT induction [37], our further analyses were focused on TGFβ1/SMAD signalling target genes, including the transcription factors SNAIL and SLUG, which in turn induced the expression of mesenchymal genes (such as Vimentin and α-SMA) [38, 39] and the repression of epithelial marker genes (such as E-cadherin and CK8/18). Western blot analyses showed that CRC_sEVs elicited effects in hepatocytes due to the activation of the TGFβ1/SMAD signaling pathway with different time courses for the different analyzed target genes. As reported in Fig. 6b, the significantly and early upregulated targets were the transcription factors SNAIL (after 6 h) and SLUG (after 12 h). Moreover, the mesenchymal marker Vimentin started to be significantly modulated from 6 h, according to data in the literature describing it as an early target gene of the TGFβ1/SMAD signaling pathway [40]. The modulation of Vimentin was then appreciable until 48 h, when we found that α-SMA was also significantly increased (Fig. 6c). Finally, significant repression of the epithelial markers CK8/18 and E-cadherin was detectable after 48 h of CRC_sEV treatment (Fig. 6d). Together, these findings suggest that CRC_sEVs elicited the activation of TGFβ1/SMAD signalling in hepatocytes associated with the expression of early and late EMT markers.

According to what we observed with sEVs isolated from SW480 and SW620 cells, we found that sEVs isolated from the plasma of CRC patients (CRC_P/sEVs) were able to activate the expression of the EMT transcription factors SNAIL and SLUG as well as Vimentin. In detail, the results reported in Fig. 6e show that CRC_P/sEVs, but not sEVs isolated from the plasma of CRC patients (HS/sEVs), induced the upregulation of SNAIL, SLUG, and Vimentin after 6 h of treatment in hepatocytes. As specified above, since the Western blot assays of CRC_P/sEV- and HS/sEV-treated hepatocytes were also performed separately, we paid attention to loading the same protein concentration and fixing the same exposure time for protein detection. The two membranes stained with Ponceau showing the same protein loading are reported in (Additional File 8: Figure S5).

**Fig. 7:**
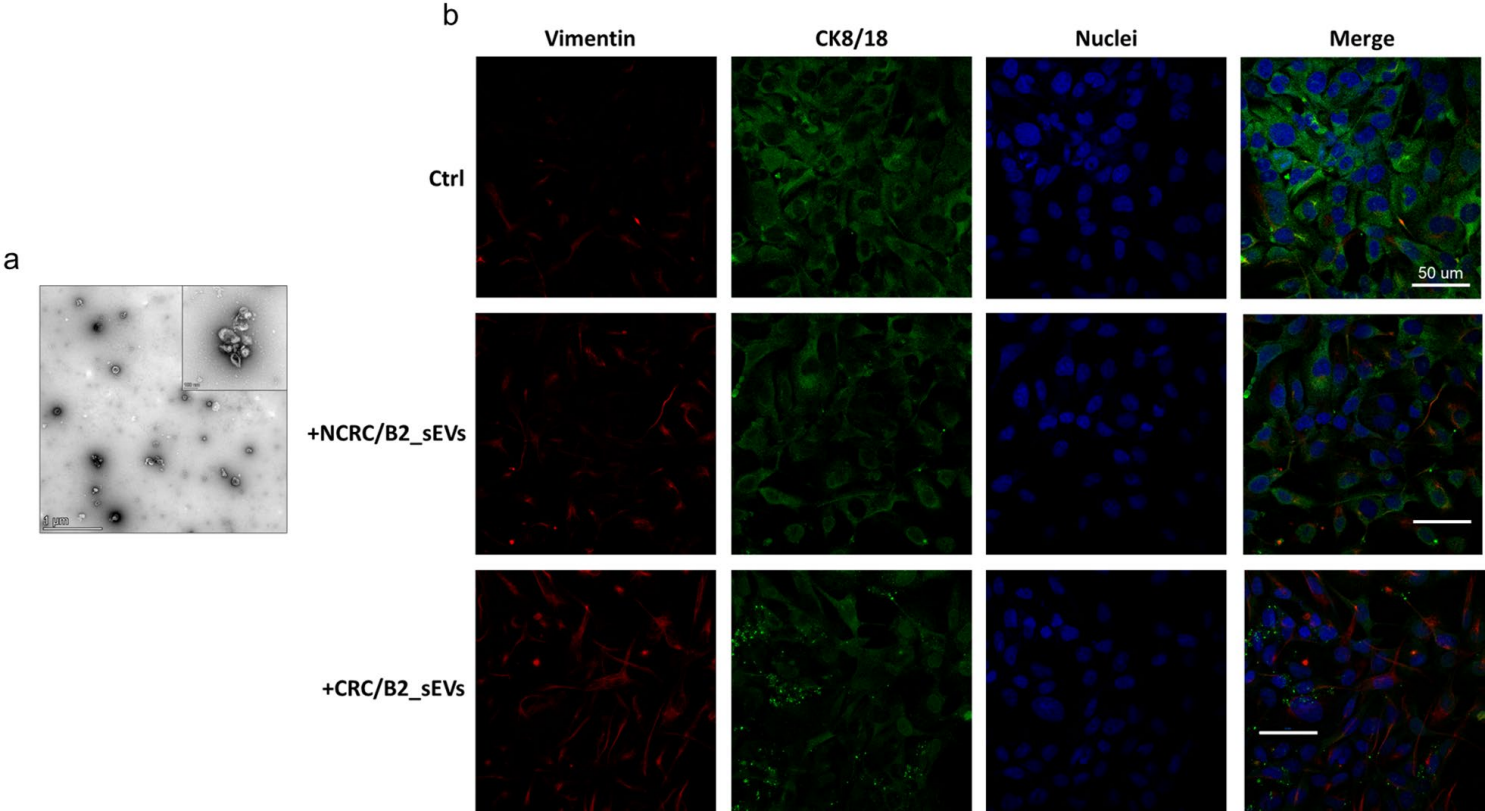
sEVs isolated from CRC biopsies modulate the expression of mesenchymal and epithelial markers in hepatocytes. **a** Representative TEM micrograph of sEVs isolated from CRC biopsy (CRC/B1 in Table 2). Scale bars: 1 µm and 100 nm. **b** Confocal microscopy analysis of Vimentin (red) and CK8/18 (green) in hepatocytes treated with sEVs isolated from a CRC biopsy (CRC/B2 in Table 2; CRC/B2_sEVs) and from the corresponding non-CRC mucosa (NCRC/B2 in Table 2; NCRC/B2_sEVs) for 24 h; nuclei are in blue. Untreated cells are indicated as control (Ctrl)

Interestingly, we found that the treatment of hepatocytes for 24 h with sEVs isolated from CRC biopsy (a representative TEM image of these sEVs is reported in Fig. 7a) induced a concurrent increase in Vimentin and a decrease in CK8/18 expression, unlike sEVs isolated from the biopsy of the corresponding non-CRC mucosa (Fig. 7b).

## Discussion

Accumulating evidence suggests that EVs released by primary tumor cells are key players in the regulation of the metastatic process, including the early step driving the formation of the PMN [11, 41–43]. TD_sEVs can reach and alter secondary sites before tumor cell arrival, conditioning the PMN via immunosuppression, macrophage polarization, angiogenesis, stromal cell remodeling, and oncogenic reprogramming [43–46]. In the liver, it has been described that TD_sEVs lead to the formation of a fibrotic and immunosuppressive PMN directly targeting Kupffer cells, hepatic stellate cells, and NK cells, while no data are available about the involvement of hepatocytes [8, 11, 47]. In this study, we provided the first evidence that CRC-derived sEVs directly affect the phenotypic traits of hepatocytes. We found that CRC_sEVs carrying TGFβ1 elicit a decrease in hepatocyte marker genes (HNF4, albumin, APOE, and CYP3A4) and the activation of a TGFβ1/SMAD-dependent EMT associated with the acquisition of mesenchymal markers (Vimentin and a-SMA) and with a reduction in epithelial marker expression (CK8/18 and E-cadherin). Our findings suggest a new role of TGFβ1-carrying CRC_sEVs in inducing an early alteration of structural and functional properties of liver parenchyma, thus anticipating the liver damage often associated with metastasis and leading hepatocytes to EMT described as an early step of fibrogenesis [16].

TGFβ1 is reported as a central player in liver injury and is able to induce a decrease in several adult hepatocyte markers, such as HNF-4α and albumin, thus affecting hepatic-specific functions [31, 32]. Moreover, TGFβ1 is described as a stronger signal that can regulate cell plasticity, inducing EMT-associated modifications of different liver cell populations, including hepatic stellate cells and hepatocytes, thus contributing to liver fibrosis [15, 16, 48]. Hepatic fibrosis is a non-physiological process characterized by excessive extracellular matrix deposition, which causes tissue damage and failure or alteration of proper liver function. It is considered a key driver of chronic liver injury, and the fibrosis niche is described as a favorable microenvironment for metastatic formation in the liver [13]. Moreover, recent studies have reported that liver fibrosis is a powerful negative predictor of hepatic-specific disease-free survival and relapse-free survival in CRC [12]. Even if hepatic stellate cells are widely considered the main matrix-producing cells that drive liver fibrosis, evidence from several in vitro and in vivo studies suggests that TGFβ1-stimulated hepatocytes can undergo phenotypic and functional changes and can acquire a fibroblast-like morphology, leading to EMT associated with liver fibrosis (49 and references therein). However, since some contradictory results have questioned the EMT of hepatocytes and its contribution to liver fibrogenesis [50], the debate is still open, and numerous studies have focused on this topic [51]. Recently, it has been reported that during the pro-metastatic process, hepatocytes actively participate to alter the immune and fibrotic microenvironment of the liver, inducing the activation of IL-6/STAT3 signaling and the subsequent production of serum amyloid A1 and A2 [52]. Our study, demonstrating that CRC_sEVs elicit hepatocytes to undergo EMT, points out an early potential activation of their pro-fibrotic behavior that can contribute to shaping an environment supporting tumor cell colonization. Our next work will focus on unveiling the molecular mechanisms through which the CRC_sEV-mediated EMT of hepatocytes can contribute to the formation of a fibrotic and tumor-supportive microenvironment [14]. Since metastasis is a complex mechanism in which many other factors are involved (e.g., organotropism, specific uptake in the organ, modulation of several cell phenotypes), further studies using in vivo models will be mandatory to assure the implication of CRC sEVs in determining the involvement of hepatocytes during liver pre-metastatic niche formation and the related consequences.

## Conclusions

In conclusion, our study revealed that CRC_sEVs directly target hepatocytes, triggering TGFβ1-mediated EMT, suggesting for the first time that CRC_sEV-educated hepatocytes may have an active role in the early stage of CRC liver metastasis formation. This new evidence may offer new insights to develop more effective targeted therapeutic approaches against the formation of hepatic metastases.

## Supplementary Information

**Additional file 1: **NTA VIDEOS of SW80_sEVS – Loaded separately

**Additional file 2: **NTA VIDEOS of SW620_sEVs – Loaded separately.

**Additional file 3: Figure S1: **Confocal micrographs showing the time-dependent uptake of CRC_sEVs into hepatocytes.

**Additional file 4: Figure S2.** NTA showed that treatment with 0.125% trypsin for 15 minutes at 37°C did not affect the integrity of the SW480_sEVs and SW620_sEVs.

**Additional file 5: **NTA VIDEO of CRC_P/Plasma_sEVs (P2) – Loaded separately

**Additional file 6: Figure S3.** Ponceau-S-stained nitrocellulose membrane used for the Western blots reported in Figure 6c. The proteins of sEVs isolated from 5 CRC patient plasma samples (P1-5; CRC_P/Plasma_sEVs) or 5 healthy subject plasma samples (HS1-5; HS/Plasma_sEVs) were loaded in each lane.

**Additional file 7: Figure S4**. CellTox assay showed that treatment for 24 and 48 h with CRC_P/sEVs (a) and HS/sEVs (b) did not alter the viability of hepatocytes (RFU: relative fluorescence units).

**Additional file 8: Figure S5.** Ponceau-S-stained nitrocellulose membrane used for the Western blots reported in Figure 6d. The protein extract loaded in each lane was obtained from hepatocytes treated for 6 h with SEVs isolated from 5 CRC patient plasma samples (P1-5; CRC_P/Plasma_sEVs) or 5 healthy subject plasma samples (HS1-5; HS/Plasma_sEVs); Ctrl: untreated control cells.

## Data Availability

All data related to this study are included in this paper as Supplementary Information. During the current study, no datasets were generated or analyzed. On reasonable request, further experimental details will be provided by the corresponding author.

## Abbreviations

ALBU: Albumin
APOE: Apolipoprotein E
BSA: Albumin from bovine serum
CK: Cytokeratin
CRC: Colorectal cancer
CRC_P: Plasma of CRC patients
CRC_sEVs: SEVs derived from CRC cells
CRC/B: CRC biopsy
CYP3A4: Cytochrome P450 3A4
EGF: Epidermal growth factor
EMT: Epithelial to mesenchymal transition
FBS: Fetal bovine serum
HNF4: Hepatocyte nuclear factor 4
HRP: Horseradish peroxidase
HS/sEVs: SEVs derived from healthy subjects
MIF: Migration inhibitory factor
NCRC/B: Non-colorectal cancer biopsy
NTA: Nanoparticle tracking analysis
PMN: Pre-metastatic niche
RT-PCR: Reverse transcription quantitative polymerase chain reaction
sEVs: Small extracellular vesicles
αSMA: Alpha smooth muscle actin
TD_EVs: Tumor-derived extracellular vesicles
TEM: Transmission electron microscope
TGFβ1: Transforming growth factor β1
THLE-2: T antigen-immortalized healthy human liver epithelial
