# Supplementary figures and images for: Colorectal cancer-derived small extracellular vesicles induce TGFβ1-mediated epithelial to mesenchymal transition of hepatocytes
Source: Cancer Cell Int. 2023 Apr 18;23:77. doi: 10.1186/s12935-023-02916-8 (PMC10114452; doi:10.1186/s12935-023-02916-8)

## Slide 1
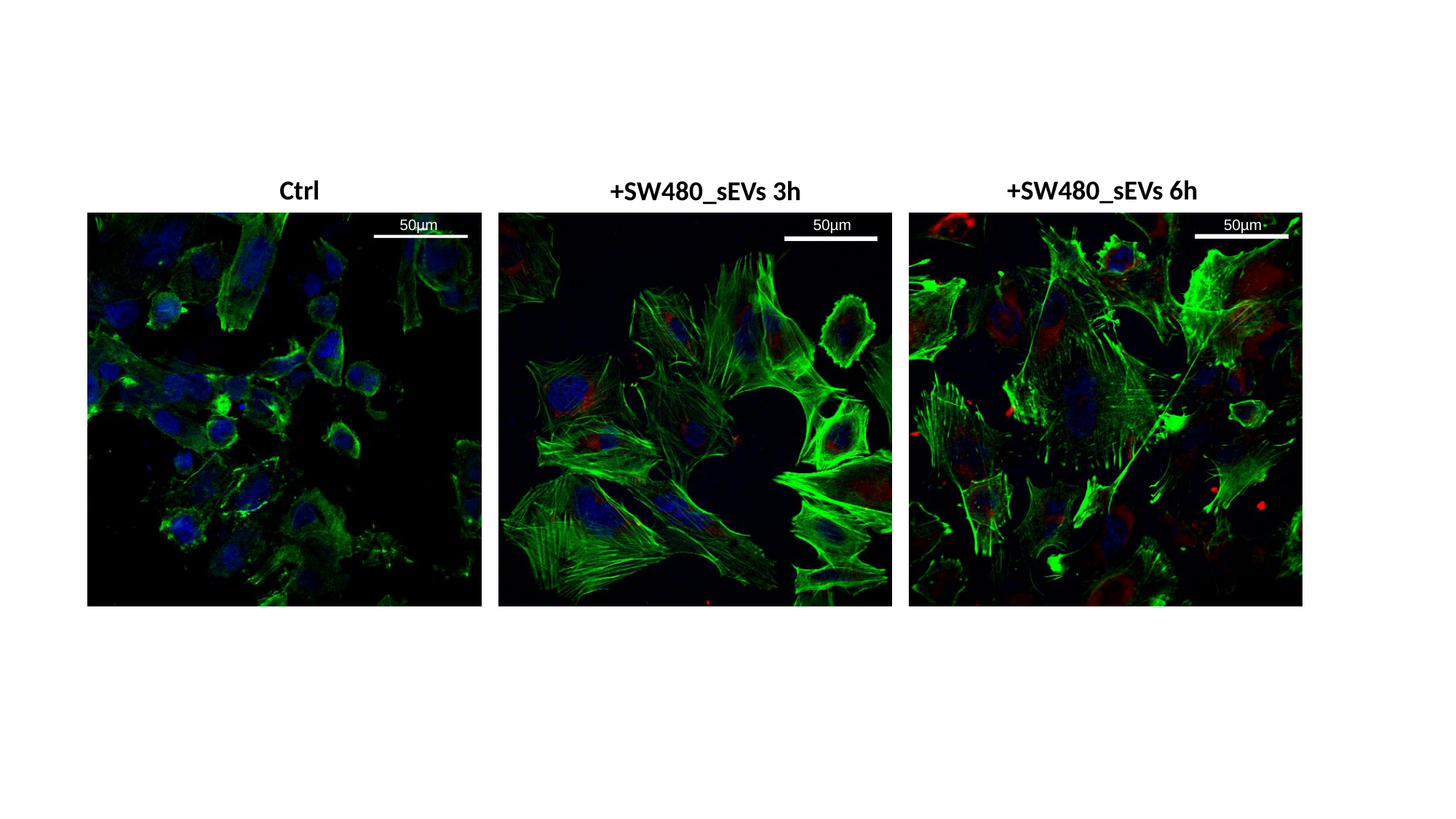

+SW480_sEVs 6h
Ctrl
+SW480_sEVs 3h
50µm
50µm
50µm

Supplement: Supplementary file 3 — Additional file 3: Figure S1: Confocal micrographs showing the time-dependent uptake of CRC_sEVs into hepatocytes. [file 12935_2023_2916_MOESM3_ESM.pptx]

## Slide 1
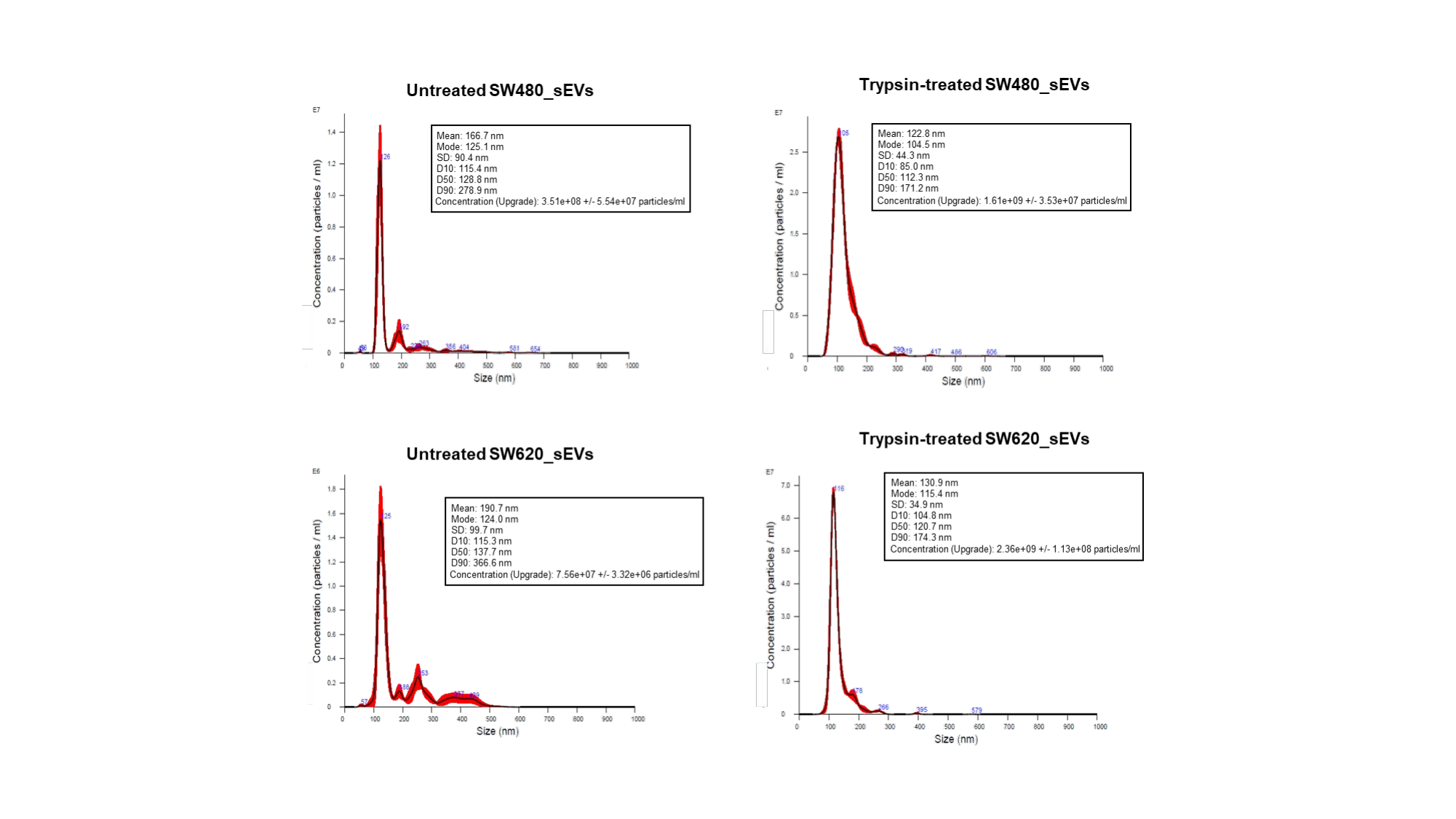

Supplement: Supplementary file 4 — Additional file 4: Figure S2. NTA showed that treatment with 0.125% trypsin for 15 minutes at 37°C did not affect the integrity of the SW480_sEVs and SW620_sEVs. [file 12935_2023_2916_MOESM4_ESM.pptx]

## Slide 1
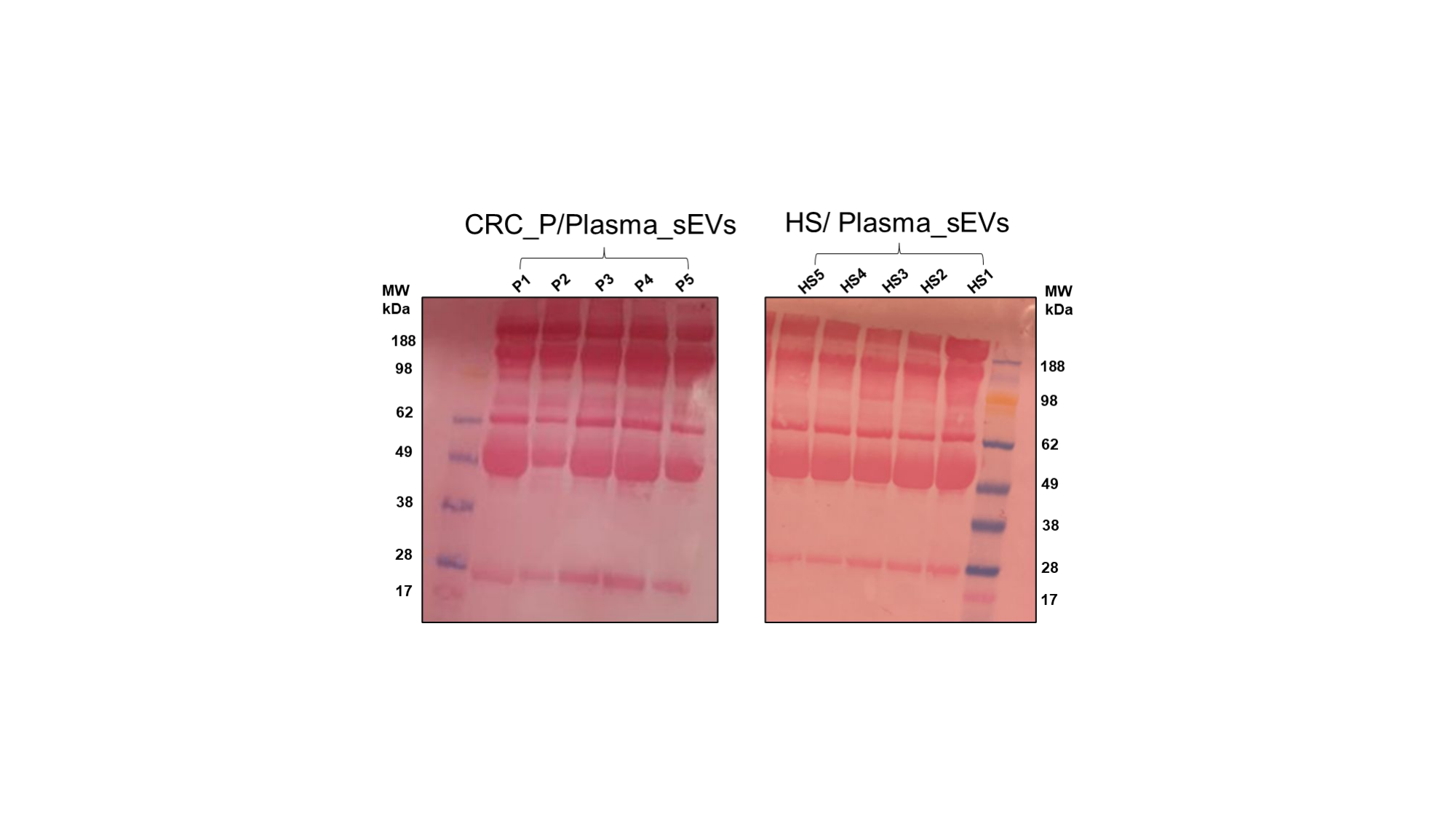

Supplement: Supplementary file 6 — Additional file 6: Figure S3. Ponceau-S-stained nitrocellulose membrane used for the Western blots reported in Figure 6c. The proteins of sEVs isolated from 5 CRC patient plasma samples (P1-5; CRC_P/Plasma_sEVs) or 5 healthy subject plasma samples (HS1-5; HS/Plasma_sEVs) were loaded in each lane. [file 12935_2023_2916_MOESM6_ESM.pptx]

## Slide 1
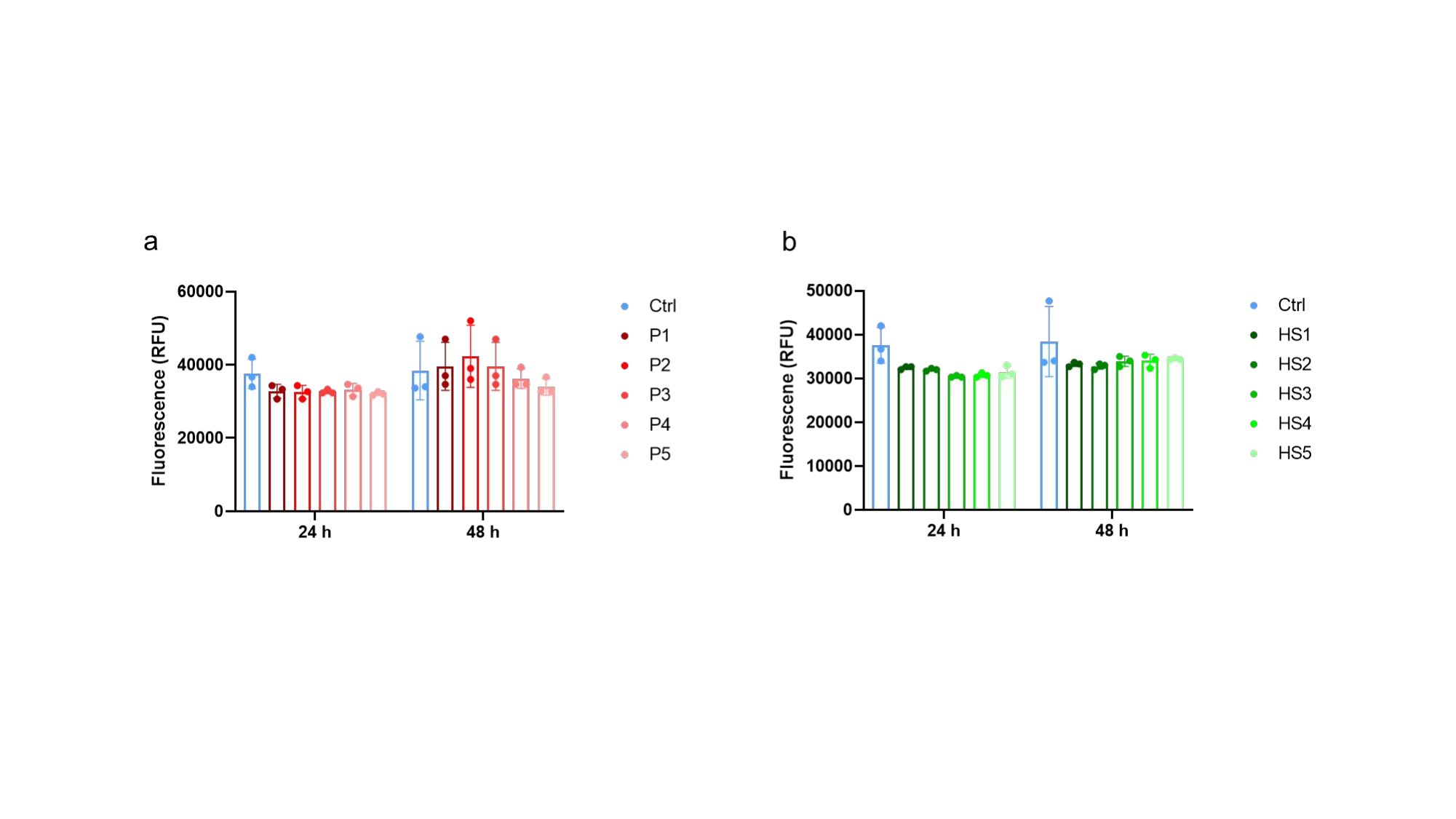

Supplement: Supplementary file 7 — Additional file 7: Figure S4. CellTox assay showed that treatment for 24 and 48 h with CRC_P/sEVs (a) and HS/sEVs (b) did not alter the viability of hepatocytes (RFU: relative fluorescence units). [file 12935_2023_2916_MOESM7_ESM.pptx]

## Slide 1
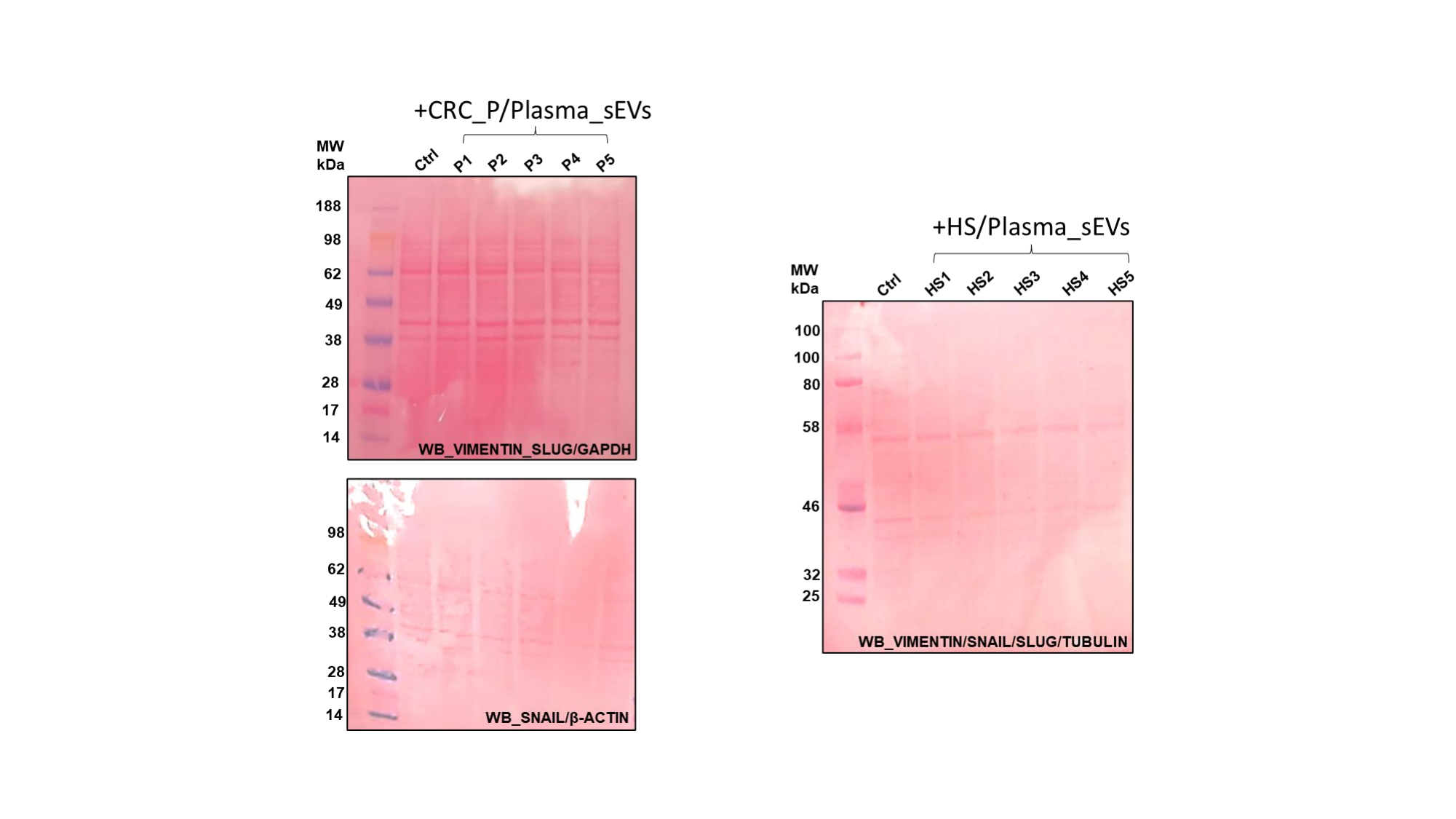

Supplement: Supplementary file 8 — Additional file 8: Figure S5. Ponceau-S-stained nitrocellulose membrane used for the Western blots reported in Figure 6d. The protein extract loaded in each lane was obtained from hepatocytes treated for 6 h with SEVs isolated from 5 CRC patient plasma samples (P1-5; CRC_P/Plasma_sEVs) or 5 healthy subject plasma samples (HS1-5; HS/Plasma_sEVs); Ctrl: untreated control cells. [file 12935_2023_2916_MOESM8_ESM.pptx]
